# Postnatal nutritional intakes and hyperglycemia as determinants of blood pressure at 6.5 years of age in children born extremely preterm

**DOI:** 10.1038/s41390-019-0341-8

**Published:** 2019-02-18

**Authors:** Itay Zamir, Elisabeth Stoltz Sjöström, Anna-Karin Edstedt Bonamy, Lilly-Ann Mohlkert, Mikael Norman, Magnus Domellöf

**Affiliations:** 1 Department of Clinical Sciences, Pediatrics, Umeå University, Umeå, Sweden; 2 Department of Food and Nutrition, Umeå University, Umeå, Sweden; 3 Department of Women’s and Children’s Health, Karolinska Institutet, Stockholm, Sweden; 4 Clinical Epidemiology Unit, Department of Medicine Solna, Karolinska Institutet, Stockholm, Sweden; 5 Sachs’ Children’s and Youth Hospital, Södersjukhuset, Stockholm, Sweden; 6 Department of Clinical Science, Intervention and Technology, Karolinska Institutet, Stockholm, Sweden; 7 Department of Neonatal Medicine, Karolinska University Hospital, Stockholm, Sweden

## Abstract

**Background:**

Adverse developmental programming by early-life exposures might account for higher blood pressure (BP) in children born extremely preterm. We assessed associations between nutrition, growth and hyperglycemia early in infancy, and BP at 6.5 years of age in children born extremely preterm.

**Methods:**

Data regarding perinatal exposures including nutrition, growth and glycemia status were collected from the Extremely Preterm Infants in Sweden Study (EXPRESS), a population-based cohort including infants born <27 gestational weeks during 2004–2007. BP measurements were performed at 6.5 years of age in a sub-cohort of 171 children (35% of the surviving children).

**Results:**

Higher mean daily protein intake (+1 g/kg/day) during postnatal weeks 1–8 was associated with 0.40 (±0.18) SD higher diastolic BP. Higher mean daily carbohydrate intake (+1 g/kg/day) during the same period was associated with 0.18 (±0.05) and 0.14 (±0.04) SD higher systolic and diastolic BP, respectively. No associations were found between infant growth (weight, length) and later BP. Hyperglycemia and its duration during postnatal weeks 1–4 were associated primarily with higher diastolic BP *z*-scores.

**Conclusions:**

These findings emphasize the importance of modifiable early-life exposures, such as nutrition and hyperglycemia, in determining long-term outcomes in children born extremely preterm.

## Introduction

Children born extremely preterm (EPT) have higher systolic and diastolic blood pressures (SBP and DBP) than term-born controls already at 2.5 and 6.5 years of age, and preterm birth has previously been linked with higher SBP later in life.^1–3^ The mechanisms behind these findings have not been fully understood. One hypothesis refers to developmental programming by early-life exposures. According to this theory, early malnutrition contributes to BP elevation later in life.^4^

Concerns have been raised that not only under- but also overnutrition in infancy might contribute to higher blood pressure and increased cardiovascular risk later in life.^5^ However, major differences in nutrient intakes from diets randomly assigned to infants <1850 g born in 1982–1985 were not followed by differences in later blood pressure.^6^ As other randomized controlled trials on different nutritional management are scarce, observational studies have focused mainly on early growth, mediated by the nutrition given, and its effects on later outcomes. Accelerated weight gain has been suggested as a possible risk factor, being associated with increased SBP in school-age children born preterm.^7^

Hyperglycemia is a specific risk factor for macro- and microvascular injuries. It is common in EPT infants during the neonatal period,^8^ but has not been investigated in relation to BP or cardiovascular disease later in life.

To improve the long-term health of survivors after preterm birth, it is important to clarify how modifiable early-life exposures affect longer term outcome. This study aimed at assessing the associations between nutrition, growth and hyperglycemia in early life and BP at 6.5 years of age in children born EPT. We hypothesized that poor neonatal nutrition and growth, as well as postnatal hyperglycemia, would be associated with higher BP at long-term follow-up.

## Methods

### Study population

The Extremely Preterm Infants in Sweden Study (EXPRESS) is a population-based cohort including all infants born at gestational age <27 completed weeks during a 3-year period from 1 April 2004 to 31 March 2007. Cohort characteristics, nutritional intakes, perinatal and growth outcomes, as well as survival and morbidity outcomes have been previously published.^9–13^ All 494 survivors were invited to a follow-up study at age 6.5 years ± 3 months. Children living in 3 of the 7 participating regions—Lund, Stockholm and Umeå—250 (50.6%) in total, were further invited to perform cardiovascular and lung function assessment, including BP measurements. In these mentioned regions, 12 children had known congenital heart or lung malformations, and were not invited to the follow-up. Out of this group, 38 children and their parents declined to participate, 7 children were lost to follow-up (either could not be traced or emigrated), and in 5 children no BP or heart rate measurements could be obtained. One child had bilateral pulmonary artery stenosis and one child had left ventricle outflow tract obstruction, which were regarded as ongoing cardiovascular diseases and their follow-up results were therefore excluded from the study. We further excluded 27 children due to follow-up visit that was not performed within the time period defined above. As previously published, there were no significant differences in mean gestational age at birth, mean birth weight and sex distribution between children included and those lost to follow-up.^2^

### Ethics

This study was approved by the regional ethics committee in Lund (no. 42/2004; original EXPRESS) and by the regional ethics committee in Stockholm (no. 520-31/2/2010, amendment no. 376-32/2011; follow-up study). All the parents or legal guardians of the participating children have given written informed consent.

### Exposures and covariates

Perinatal clinical data in infancy were extracted from the EXPRESS cohort database. Nutritional and anthropometric data were retrospectively collected as previously described.^8,11,14^ Daily data were collected from hospital records regarding enteral and parenteral intakes of macronutrients (protein, carbohydrates and fat; g/kg/day) until postnatal day 28. Thereafter, data were registered once per week, on postnatal days 35, 42, etc. Enteral and parenteral intakes were added together. Manufacturer data and breast milk analyses results were used to calculate nutrient intakes. Routine analyses of the infants’ own mother or donated breast milk were performed at most hospitals using infrared analysis (Milkoscan 4000, Eurofins Steins Laboratory AB, Jönköping, Sweden). In 32% of the infants, breast milk samples were not analyzed and were assumed to have average content of the analyzed samples. The intakes included fluids from drug infusions, flush solution, etc. Nutritional and biochemical markers data were collected and stored using a computerized system (Nutrium software by Nutrium AB, Umeå, Sweden). Growth data included length and weight measurements, as well as the respective *z*-scores, at birth, and on postnatal days 28 and 56. When data were missing, they were interpolated linearly based on existing data.

Collection of data regarding glycemia status during the first 28 postnatal days was previously described.^8^ It included recording all available plasma glucose measurements, where the vast majority of them were analyzed in plasma samples using blood gas analyzer (most often: Radiometer, Brønshøj, Denmark). Measurements were excluded when samples were taken from a venous line with an ongoing glucose infusion.

### Outcomes

The procedure for BP measurements was previously described.^2^ In short, families were invited to attend a follow-up visit during morning time. Height and weight of the child were measured. SBP and DBP were measured in  the right arm which was placed at heart’s level while the child was sitting and after 15 min of rest in the examination room, using a validated oscillometric device (Omron HEM 907; Omron Healthcare, Kyoto, Japan). Three BP measurements were taken with at least 2-min intervals and mean values were calculated. In cases where only two BP measurements could be obtained, the mean value was calculated using these values. When only one BP measurement was obtained, this value was used instead of a mean value. SBP and DBP *z*-scores were calculated using age-, sex-, and height-adjusted BP coefficients for children.^15^ *z*-Scores for current height were adjusted for age and sex and were calculated using the World Health Organization growth reference data from 2007 for ages 5–19 years.^16^

### Statistical analyses

Data were analyzed using SPSS Statistical software (IBM Corp. Released 2016. IBM SPSS Statistics for Windows, Version 24.0. Armonk, NY: IBM Corp.). Possible associations between early nutritional intakes and BP measurements were tested for in multiple linear regression models. Separate analyses were performed for the total intake of each macronutrient (protein, carbohydrates and fat) given to the children during postnatal weeks 1–4, 5–8, and 1–8. Blood products, such as transfused erythrocytes and plasma, were not taken into account in these analyses.

The changes in weight, length and *z*-scores were calculated for the time periods mentioned above. Multiple linear regression models were used to determine possible associations between the growth parameters and BP measurements.

Multiple linear regression models were also used to analyze possible associations between the duration (in days) of hyperglycemia occurring during postnatal weeks 1–4 and later BP measurements. Analyses of covariance (ANCOVA) were conducted to determine the difference in BP measurements between children who had early-life hyperglycemia and those who did not. These analyses were done with and without mean daily carbohydrate intake during postnatal weeks 1–4 as a cofactor. In these analyses, different definitions for hyperglycemia were used, both regarding the threshold of glucose concentrations (>180, 216 and 252 mg/dL [10, 12 and 14 mmol/L]) as well as different durations (2 or 3 consecutive days). An infant was regarded as hyperglycemic on a specific day if the highest glucose measurement recorded on that day was higher than the respective threshold.

According to previously published results from this cohort regarding the determinants of SBP and DBP *z*-scores,^2^ all models in the study were adjusted for gestational age at birth and heart rate at follow-up. The analyses were not adjusted for postnatal steroid treatment or weight and body mass index at follow-up since these factors were not found to be associated with BP in preliminary analyses. The significance level was set to *P* *<* 0.05.

## Results

In total, 171 children were included in the analysis. Triplicate, duplicate and single BP measurements were obtained in 95%, 3% and 2% of the children, respectively. Participant characteristics are described in Table 1. Data for anthropometry measures and nutrition intakes given during the study period, as well as blood pressure and heart rate measurements at follow-up, are presented in Supplemental Tables S1 and S2 (online). As previously reported, lower gestational age at birth was associated with higher SBP and DBP *z*-scores, but not with other perinatal covariates, such as corticosteroid treatment before or after birth, or smoking during pregnancy.^2^

**Table 1:** Participant characteristics

	Number (%)
Children included	171
Males	95 (56)
Smoking during pregnancy	18 (11)
Preeclampsia	17 (10)
Antenatal corticosteroid treatment	150 (88)

	Mean (SD)
Maternal age at birth, years	31.5 (5.4)
Maternal body mass index at birth, kg/m^2^	22.7 (9.3)
Gestational age at birth, weeks	25.4 (1.0)
Duration of postnatal corticosteroid treatment, days	5 (9.3)

### Early nutrition and later BP

#### Protein intake

Mean daily protein intakes during postnatal weeks 1–4 and 5–8 were not significantly associated with either SBP or DBP *z*-scores at follow-up (Table 2; results not shown for postnatal weeks 5–8). Mean daily protein intake given during the first 8 postnatal weeks was significantly associated with DBP *z*-score—an increase of 1 g/kg/day in the protein intake during this period was associated with an increase of 0.40 standard deviations (SD) in the DBP at follow-up (*P* = 0.028).

**Table 2:** Multiple linear regression models assessing nutritional intakes during the first 8 postnatal weeks as predictors of blood pressure at 6.5 years of age in 171 extremely preterm-born children (adjusted for gestational age at birth and heart rate measured at follow-up)

	*z*-SBP		*z*-DBP	
	*β* (95% CI)	*P* Value	*β* (95% CI)	*P* Value
Mean daily protein intake, g/kg/day				
Postnatal weeks 1–4	0.14 (−0.22, 0.50)	0.44	0.23 (−0.07, 0.54)	0.13
Postnatal weeks 1–8	0.37 (−0.08, 0.82)	0.11	0.40 (0.04, 0.76)	**0.03**
Mean daily carbohydrate intake, g/kg/day				
Postnatal weeks 1–4	0.14 (0.06, 0.23)	**0.001**	0.12 (0.04, 0.19)	**0.002**
Postnatal weeks 1–8	0.18 (0.08, 0.28)	**0.001**	0.14 (0.06, 0.23)	**0.001**
Mean daily lipid intake, g/kg/day				
Postnatal weeks 1–4	0.00 (−0.11, 0.11)	0.97	−0.04 (−0.14, 0.05)	0.34
Postnatal weeks 1–8	−0.03 (−0.14, 0.09)	0.67	−0.05 (−0.14, 0.04)	0.30

Statistically significant values are in bold *p* < 0.05

#### Carbohydrate intake

Increased mean daily carbohydrate intakes during postnatal weeks 1–4, 5–8 and 1–8, were significantly associated with increased *z*-score for both SBP and DBP at follow-up. For example, an increase of 1 g/kg/day in the mean daily carbohydrate intake during the first 8 postnatal weeks was associated with increases of 0.18 SD in SBP and 0.14 SD in DBP at follow-up (*P* = 0.001 for both).

#### Fat intake

Mean daily lipid intakes during postnatal weeks 1–4, 5–8 and 1–8 were not significantly associated with either SBP or DBP *z*-scores at follow-up.

### Early growth and later BP

Shorter length on postnatal days 28 and 56 was significantly associated with higher DBP *z*-scores at follow-up (Table 3). However, no significant associations were found between change in length during the first 8 postnatal weeks and later BP *z*-scores (Table 4; results for postnatal weeks 5–8 not shown). No significant associations were found between weight or change in weight during the first 8 postnatal weeks and later BP *z*-scores.

**Table 3:** Multiple linear regression models assessing anthropometry measures during the first 8 postnatal weeks as predictors of blood pressure at 6.5 years of age in 171 extremely preterm-born children (adjusted for gestational age at birth and heart rate measured at follow-up)

	*z*-SBP		*z*-DBP	
	*β* (95% CI)	*P* Value	*β* (95% CI)	*P* Value
At birth				
Weight, 100 g	−0.03 (−0.11, 0.06)	0.50	−0.06 (−0.13, 0.01)	0.08
Weight *z*-score, SD	−0.03 (−0.12, 0.06)	0.54	−0.07 (−0.15, 0.01)	0.08
Length, cm	0.01 (−0.05, 0.07)	0.81	−0.04 (−0.09, 0.01)	0.08
Length *z*-score, SD	0.01 (−0.07, 0.08)	0.87	−0.05 (−0.12, 0.01)	0.10
On postnatal day 28				
Weight, 100 g	−0.03 (−0.11, 0.05)	0.43	−0.04 (−0.10, 0.02)	0.21
Weight *z*-score, SD	−0.05 (−0.15, 0.06)	0.39	−0.04 (−0.13, 0.04)	0.32
Length, cm	−0.02 (−0.09, 0.04)	0.48	−0.07 (−0.12, −0.02)	**0.01**
Length *z*-score, SD	−0.04 (−0.13, 0.05)	0.37	−0.09 (−0.16, −0.02)	**0.01**
On postnatal day 56				
Weight, 100 g	0.01 (−0.04, 0.05)	0.80	−0.02 (−0.05, 0.02)	0.41
Weight *z*-score, SD	0.01 (−0.08, 0.10)	0.87	−0.03 (−0.10, 0.05)	0.44
Length, cm	−0.01 (−0.07, 0.04)	0.62	−0.05 (−0.09, −0.002)	**0.04**
Length *z*-score, SD	−0.02 (−0.10, 0.06)	0.59	−0.06 (−0.13, 0.00)	0.05

Statistically significant values are in bold *p* < 0.05

**Table 4:** Multiple linear regression models assessing growth parameters as predictors of blood pressure at 6.5 years of age in 171 extremely preterm-born children (adjusted for gestational age at birth and heart rate measured at follow-up)

	*z*-SBP		*z*-DBP	
	*β* (95% CI)	*P* Value	*β* (95% CI)	*P* Value
Postnatal weeks 1 to 4				
Weight change, 100 g	−0.02 (−0.14, 0.10)	0.77	0.02 (−0.08, 0.12)	0.71
Weight *z*-score change, SD	−0.02 (−0.17, 0.13)	0.80	0.08 (−0.05, 0.21)	0.20
Length change, cm	−0.02 (−0.14, 0.10)	0.75	0.03 (−0.06, 0.13)	0.50
Length *z*-score change, SD	−0.04 (−0.18, 0.11)	0.62	0.04 (−0.08, 0.16)	0.54
Postnatal weeks 1 to 8				
Weight change, 100 g	0.02 (−0.04, 0.08)	0.44	0.00 (−0.05, 0.05)	0.97
Weight *z*-score change, SD	0.05 (−0.06, 0.17)	0.35	0.05 (−0.05, 0.14)	0.30
Length change, cm	0.02 (−0.08, 0.12)	0.70	−0.03 (−0.11, 0.05)	0.48
Length *z*-score change, SD	0.02 (−0.12, 0.15)	0.81	−0.03 (−0.14, 0.08)	0.60

### Early hyperglycemia and later BP

Neonatal hyperglycemia >216 mg/dL (12 mmol/L) for 2 consecutive days was significantly associated with higher SBP *z*-scores at follow-up than in children who did not experience such hyperglycemia (Table 5). Neonatal hyperglycemia >180 mg/dL (10 mmol/L) for 3 consecutive days, >216 mg/dL (12 mmol/L) for 2 and 3 consecutive days, and >252 mg/dL (14 mmol/L) for 2 consecutive days was significantly associated with higher DBP *z*-score at follow-up than in children without such hyperglycemia. These results remained unchanged (except for neonatal hyperglycemia >180 mg/dL (10 mmol/L) for 3 consecutive days) when mean daily carbohydrate intake during postnatal weeks 1–4 was introduced into the model, while increased carbohydrate intake remained significantly associated with increased SBP and DBP *z*-score.

**Table 5:** Comparison of blood pressure at 6.5 years of age in 171 extremely preterm-born children between children who had hyperglycemia during the first 4 postnatal weeks and children who did not (different definitions for hyperglycemia were used; adjusted for gestational age at birth and heart rate measured at follow-up)

		*z*-SBP				*z*-DBP			
Hyperglycemia definition/Duration of episode	*N* ^a^	Adjusted mean (SE) ^a^	Adjusted mean (SE)^b^	Adjusted mean difference (95% CI)	*P* Value	Adjusted mean (SE) ^a^	Adjusted mean (SE) ^b^	Adjusted mean difference (95% CI)	*P* Value
>180 mg/dL (10 mmol/L)									
2 consecutive days	80	0.11 (0.08)	0.22 (0.08)	−0.10 (−0.34, 0.14)	0.39	−0.02 (0.07)	0.08 (0.07)	−0.10 (−0.30, 0.10)	0.34
3 consecutive days	49	0.35 (0.11)	0.10 (0.07)	0.26 (−0.01, 0.53)	0.06	0.20 (0.09)	−0.03 (0.06)	0.24 (0.01, 0.46)	**0.04**
>216 mg/dL (12 mmol/L)									
2 consecutive days	43	0.40 (0.11)	0.09 (0.06)	0.31 (0.05, 0.57)	**0.02**	0.21 (0.10)	−0.03 (0.05)	0.23 (0.01, 0.46)	**0.04**
3 consecutive days	22	0.34 (0.16)	0.14 (0.06)	0.20 (−0.14, 0.54)	0.24	0.33 (0.13)	−0.01 (0.05)	0.34 (0.06, 0.63)	**0.02**
>252 mg/dL (14 mmol/L)									
2 consecutive days	26	0.43 (0.14)	0.12 (0.06)	0.31 (−0.002, 0.62)	0.05	0.31 (0.12)	−0.02 (0.05)	0.33 (0.07, 0.59)	**0.01**
3 consecutive days	10	0.31 (0.23)	0.16 (0.06)	0.15 (−0.32, 0.63)	0.52	0.29 (0.20)	0.02 (0.05)	0.27 (−0.13, 0.67)	0.18

*SE* standard error

^a^In the hyperglycemic group

^b^In the non-hyperglycemic group

Statistically significant values are in bold *p* < 0.05

The duration (in days) of hyperglycemia >216 mg/dL (12 mmol/L) was significantly positively associated with DBP *z*-score and the duration of hyperglycemia >252 mg/dL (14 mmol/L) was significantly positively associated with both SBP and DBP *z*-scores (Table 6). Each additional day with hyperglycemia >252 mg/dL (14 mmol/L) was associated with an increase of ~0.05 SD in both SBP and DBP (*P* = 0.047 and 0.014, respectively).

**Table 6:** Multiple linear regression models assessing duration of hyperglycemia (in days) during the first 4 postnatal weeks as predictor of blood pressure at 6.5 years of age in 171 extremely preterm-born children (different definitions for hyperglycemia were used; adjusted for gestational age at birth and heart rate measured at follow-up)

Duration/definition of hyperglycemia episode	*z*-SBP		*z*-DBP	
	*β* (95% CI)	*P* Value	*β* (95% CI)	*P* Value
Duration of hyperglycemia, days				
>180 mg/dL (10 mmol/L)	0.02 (−0.01, 0.04)	0.29	0.02 (−0.003, 0.05)	0.09
>216 mg/dL (12 mmol/L)	0.03 (−0.001, 0.07)	0.05	0.04 (0.01, 0.07)	**0.009**
>252 mg/dL (14 mmol/L)	0.05 (0.00, 0.10)	**0.047**	0.05 (0.01, 0.09)	**0.01**

Statistically significant values are in bold *p* < 0.05

## Discussion

In this study, we made three important findings, which we believe may have implications for neonatal nutritional management and follow-up of children born EPT. First, we found that increased protein intake during the first 8 postnatal weeks was associated with higher DBP; second, increased carbohydrate intake during the first 8 postnatal weeks was associated with higher SBP and DBP; and third, the presence of neonatal hyperglycemia and its duration were associated with higher SBP and DBP at follow-up at 6.5 years of age. In contrast, growth (both in weight and in length) during the first 8 postnatal weeks was not associated with later BP.

We found a positive association between protein intake during the first 8 postnatal weeks and DBP *z*-score at 6.5 years of age. In a randomized study done by Singhal et al., small for gestational age infants born at term who received protein-enriched formula had higher DBP than children who received standard formula, thereby indicating a possible adverse effect of relative “protein overload” in infancy on long-term cardiovascular disease risk.^5^ Lucas et al. did not find differences in BP at 7.5–8 years of age in children born preterm who were randomized to receive different nutritional intakes.^6^ However, results from the same cohort showed that children given the nutrient-enriched diet might have had higher levels of insulin resistance.^17^ Results from a randomized trial of protein-energy supplementation vs. low energy/no protein supplementation during pregnancy and early childhood published by Webb et al. found no effect of protein-energy supplementation on BP in adulthood.^18^ In another study, no significant differences were found in BP between adolescents born preterm who received low intake of protein (>2.5 g/kg/day for <5 days) and those who received high intake of protein (>2.5 g/kg/day for ≥5 days) during the first 14 days of life.^19^ Different population samples were investigated in the different studies, which might contribute to difference in results. Infants born EPT may also be more vulnerable to the effects of early nutritional influences.

In our study, a positive association was found between carbohydrate intake during postnatal weeks 1–4, 5–8 and 1–8, and SBP and DBP *z*-scores at 6.5 years of age. This finding has not been previously described in the population of preterm-born children. In a cross-sectional study by Kell et al., increased consumption of added sugars by 7–12-year-old children was associated with increased DBP at the same age.^20^ However, in another study by Baranowski et al., time of introduction of high fat and high carbohydrate foods were not related to BP.^21^

We report no significant associations between weight parameters and later BP. Conflicting results have been reported previously. Whincup et al. reported that birth weight was inversely related to both SBP and DBP at 9–11 years of age.^22^ SBP rose more rapidly between 5–7 and 9–11 years of age in children of lower birth weight. A meta-analysis has concluded that there is an inverse linear association between birth weight and later risk of higher SBP.^23^ However, these results stem primarily from studies of term-born cohorts. A meta-analysis combining data from 9 cohorts, including 1571 adults born very low birth weight, found no association between birth weight *z*-score and BP.^24^ In a large, multi-center, longitudinal study including 911 children born with low birth weight, an increase in weight *z*-score from term to 12 months was associated with higher SBP at 6.5 years of age.^7^ These results were not adjusted for the child’s height at follow-up. Furthermore, these results did not account for weight changes during the immediate postnatal period, before term age. Vohr et al. reported a positive association between weight gain velocity between birth and 36 months of age (but not between birth and 18 months of age) and BP at 16 years of age in adolescents born extremely low birth weight.^25^ However, previously published data from our cohort reported that catch-up growth from 36 weeks’ postmenstrual age to follow-up at 2.5 years of age was inversely correlated with *z*-scores for SBP and DBP.^1^

Other studies which investigated the relationship between birth weight and later BP in children born prematurely reported similar findings to ours. Kowalski et al. have not found an association between birth weight *z*-score and BP in adolescents born EPT.^26^ Similarly, Doyle et al. reported that no significant association was found between birth weight *z*-score and BP in 18-year-old adolescents born very low birth weight.^27^ Keijzer-Veen et al. did not find an association between birth weight and BP in 19-year-olds born very preterm.^28^ In another study that included 694 children born with low birth weight, birth weight *z*-score was not associated with higher BP at 6.5 years of age.^29^

In our study, shorter length during the first postnatal months was shown to be associated with higher DBP, but linear growth was not. In a small study that included 38 children born with very low birth weight, SBP at 2 years of age was significantly associated with increments in length and length *z*-scores between 6 and 12 months of age, as well as between birth and 2 years of age.^30^ Gaskin et al. reported that stunted children (height *z*-score <−2) at 9–24 months of age had higher SBP at 7–8 years of age, while birth weight was not a significant predictor of SBP.^31^

We present a novel finding of an association between neonatal hyperglycemia and higher BP later in life, primarily DBP, independent of carbohydrate intake. It seems from our results that this association reflects a combination of the threshold used to define hyperglycemia and the duration of the hyperglycemia. To the best of our knowledge, this is the first time that such an association is reported in an EPT-born population.

Extreme prematurity has been previously suggested to be associated with altered arterial hemodynamics, primarily affecting the resistance vessels.^32^ The possible mechanisms that explain our results are probably multifactorial and more complex than is currently known. Lower functional skin capillary density and lower kidney volumes and number of nephrons have been suggested as determinants of BP in children born preterm, but were not found to be correlated with higher BP.^33,34^ Individuals born preterm experience an enhanced antiangiogenic state, which might affect BP via capillary rarefaction.^35^

Mice models have shown that early postnatal nutrition has a long-term effect on the appearance of hypertension, suggesting vascular impairment.^36^ Rat models have shown that although overfeeding enhances postnatal nephrogenesis, elevated BP and glomerulosclerosis are still observed in adults, indicating that other factors, rather than glomerular number reduction, are implicated in the pathogenesis of increased BP.^37^ It is possible that increased protein and carbohydrate intake lead to a hyperfiltration state in the kidneys, with glomerulosclerosis as a result, which in turn leads to increased BP via activation of the renin–angiotensin system.

It has been shown in rat models that glucose induces pressor effects, first through an increase of efferent sympathetic discharges and then through activation of the renin–angiotensin system.^38^ Sustained hyperglycemia has been shown to activate the renin–angiotensin system in adults diagnosed with insulin-dependent diabetes mellitus, thereby increasing systemic and renal vasomotor tone.^39,40^ This in turn might lead later to increased BP.

The strengths of this study include its prospective design regarding data collection of BP measurements; its relatively large size for this population of children; and the use of the same type of oscillometric device to measure BP at all study centers, thereby decreasing the likelihood for inter-center differences in the BP measurements. The limitations of our study include its retrospective design regarding data collection of nutrition intakes, growth and glucose status data. The study was not powered from the beginning to measure the outcomes presented. Oscillometric devices that measure BP calculate the SBP and DBP based on an algorithm unique to each manufacturer. However, this method is common in clinical practice, and the device used in our study was recommended by the British hypertension society for use in research.^41^ Our study was not designed to address the pathophysiological hypotheses mentioned and further studies are needed to elucidate the mechanisms that influence blood pressure in the preterm-born infant. A possible limitation of this study might have been a bias in the frequency of glucose testing, where infants who were more ill than others might have been tested for glucose more frequently. We have though previously showed that although significant differences in glucose testing frequencies between different hospitals existed, there was no difference in the rate of hyperglycemia.^8^

This study shows that increased protein and carbohydrate intake during the first 8 postnatal weeks, as well as the occurrence and duration of neonatal hyperglycemia, is associated with higher BP at 6.5 years of age in children born EPT. Weight gain and linear growth were not associated with later BP. These findings may have multiple implications for neonatal nutritional and clinical management. It supports the theory that early-life nutrition, and specifically “overnutrition”, might be associated with long-term adverse outcomes, though not necessarily via growth pathways. It also provides evidence for the association between early-life hyperglycemia, a common condition in EPT-born infants, and later BP elevation, which calls for further research into different methods to avoid early-life hyperglycemia in EPT infants.

## Supplementary information

Supplementary Information
