# Supplementary material for: Postnatal nutritional intakes and hyperglycemia as determinants of blood pressure at 6.5 years of age in children born extremely preterm
Source: Pediatr Res. 2019 Feb 18;86(1):115–21. doi: 10.1038/s41390-019-0341-8 (PMC6760565; doi:10.1038/s41390-019-0341-8)
Supplement: Supplementary file 1 — Supplementary Information [file 41390_2019_341_MOESM1_ESM.doc]

Supplemental Table S1. Anthropometry parameters and nutrition intakes during the first 8 postnatal weeks in 171 children born extremely preterm.

|  | **At birth** | **At postnatal day 28** | **At postnatal day 56** |
| --- | --- | --- | --- |
| **Weight, *grams*** | 782 (165) | 1000 (198) | 1596 (344) |
| **Weight z-score, *SD*** | -0.7 (1.2) | -2.9 (1.1) | -2.7 (1.3) |
| **Length, *cm*** | 33.0 (2.4) | 35.5 (2.4) | 39.5 (2.7) |
| **Length z-score, *SD*** | -1.2 (1.5) | -3.5 (1.3) | -3.8 (1.5) |
|  |  | **Postnatal weeks 1 to 4** | **Postnatal weeks 1 to 8** |
| **Daily total protein intake, *g/kg*** |  | 2.6 (0.3) | 2.9 (0.3) |
| **Daily total carbohydrate intake, *g/kg*** |  | 11.2 (1.2) | 12 (1.2) |
| **Daily total lipid intake, *g/kg*** |  | 4.3 (1.1) | 5.6 (1.2) |
| **Weight change, *g*** |  | + 217 (94) | + 814 (232) |
| **Weight z-score change, *SD*** |  | -2.2 (0.8) | -2.0 (1.1) |
| **Length change, *cm*** |  | + 2.6 (1.0) | + 6.6 (1.3) |
| **Length z-score change, *SD*** |  | -2.3 (0.9) | -2.6 (1.1) |

Mean (Standard deviation) are presented

Supplemental Table S2. Blood pressure and heart beat measurements at 6.5 years of age in 171 children born extremely premature.

|  | **Mean (SD)** |
| --- | --- |
| **Systolic blood pressure, *mmHg*** | 97.8 (7.9) |
| **Systolic blood pressure, *SDS*** | 0.17 (0.75) |
| **Diastolic blood pressure, *mmHg*** | 57.1 (7.1) |
| **Diastolic blood pressure, *SDS*** | 0.03 (0.63) |
| **Heart rate, *beats per minute*** | 88 (13) |

SDS – standard deviation score
